# Humidity-Sensing Properties of an 1D Antiferromagnetic Oxalate-Bridged Coordination Polymer of Iron(III) and Its Temperature-Induced Structural Flexibility

**DOI:** 10.3390/ma14195543

**Published:** 2021-09-24

**Authors:** Sanja Burazer, Krešimir Molčanov, Ana Šantić, Teodoro Klaser, Emmanuel Wenger, Damir Pajić, Zvonko Jagličić, Jasminka Popović, Marijana Jurić

**Affiliations:** 1 Ruđer Bošković Institute, Bijenička cesta 54, 10000 Zagreb, Croatia; Sanja.Burazer@irb.hr (S.B.); Kresimir.Molcanov@irb.hr (K.M.); asantic@irb.hr (A.Š.); Jasminka.Popovic@irb.hr (J.P.); 2 Department of Physics, Faculty of Science, University of Zagreb, Bijenička cesta 32, 10000 Zagreb, Croatia; tklaser@phy.hr (T.K.); dpajic@phy.hr (D.P.); 3 CRM2 CNRS, UMR 7036, Institut Jean Barriol, Université de Lorraine, BP 70239 Vandoeuvre-lès-Nancy, France; emmanuel.wenger@univ-lorraine.fr; 4 Institute of Mathematics, Physics and Mechanics, Jadranska 19, 1000 Ljubljana, Slovenia; zvonko.jaglicic@imfm.si or; 5 Faculty of Civil and Geodetic Engineering, University of Ljubljana, Jamova 2, 1000 Ljubljana, Slovenia

**Keywords:** oxalate-bridged coordination polymers, alkyl ammonium cations, single-crystal-to-single-crystal transformations, proton conductivity, humidity-sensing property, antiferromagnetic spin chain

## Abstract

A novel one-dimensional (1D) oxalate-bridged coordination polymer of iron(III), {[NH(CH_3_)(C_2_H_5_)_2_][FeCl_2_(C_2_O_4_)]}*_n_* (**1**), exhibits remarkable humidity-sensing properties and very high proton conductivity at room temperature (2.70 × 10^−4^ (Ω·cm)^−1^ at 298 K under 93% relative humidity), in addition to the independent antiferromagnetic spin chains of iron(III) ions bridged by oxalate groups (*J* = −7.58(9) cm^−1^). Moreover, the time-dependent measurements show that **1** could maintain a stable proton conductivity for at least 12 h. Charge transport and magnetic properties were investigated by impedance spectroscopy and magnetization measurements, respectively. Compound **1** consists of infinite anionic *zig-zag* chains [FeCl_2_(C_2_O_4_)]*_n_^n^*^−^ and interposed diethylmethylammonium cations (C_2_H_5_)_2_(CH_3_)NH^+^, which act as hydrogen bond donors toward carbonyl oxygen atoms. Extraordinarily, the studied coordination polymer exhibits two reversible phase transitions: from the high-temperature phase **HT** to the mid-temperature phase **MT** at T ~213 K and from the mid-temperature phase **MT** to the low-temperature phase **LT** at T ~120 K, as revealed by in situ powder and single-crystal X-ray diffraction. All three polymorphs show large linear thermal expansion coefficients.

## 1. Introduction

Metal-organic coordination polymers, which have a wealth of multifunctional applications due to their immense structural diversity and flexibility, are currently being extensively studied. Multifunctional properties of these materials can be achieved by combining the intrinsic properties of the host, especially the magnetic ones, with additional functionalities derived from the selected guest molecules [1,2]. The oxalate group, C_2_O_4_^2−^, certainly represents an excellent tailoring tool due to its different coordination modes to metal centers, as well as its ability to mediate magnetic interactions between paramagnetic metal ions, as evidenced by a large number of oxalate-based transition metal species of different nuclearity, connectivity, and dimensionality, many of which possess tunable magnetic frameworks [3,4,5].

The importance of high proton conductivity in solid materials for use as humidity sensors, membranes in water electrolyzers, and especially in fuel cells was recognized some time ago, but achieving it remains a challenging task [6]. Recently, coordination polymers and metal–organic frameworks providing additional proton-conducting pathways were established, thereby opening new avenues for improving proton conductivity [7,8,9,10,11]. These systems can generally be obtained by introducing (*i*) the guest molecules (such as water) and counterions or acids into the voids, which helps to create intricate hydrogen-bonded networks and, thus, improve the proton conductivity, or (*ii*) other functional groups, such as –COOH, –PO_3_H, –SO_3_H, and –OH, which can better the acidity and hydrophilicity of the organic ligands and, thus, form an efficient proton transport pathway [8].

In particular, it has been shown that bifunctional molecular materials, which possess high conductivity in addition to magnetic properties, can be achieved on a two- (2D) or three-dimensional (3D) oxalate-bridged coordination polymer platform. The formation of the 3D oxalate-based anionic networks with the original topologies and an unusual Mn^II^/Cr^III^ ratio leads to the appearance of ferro- and antiferromagnetic long-range order, while the presence of water, ammonium, or imidazolium molecules within the channels enables high humidity-dependent proton conductivity of these compounds [12,13,14]. It has been shown that the proton conductivity of layered 2D oxalate-bridged [M^II^Cr^III^] (M^II^ = Fe, Mn) frameworks with carboxyl group-bearing cationic components can be improved by increasing the hydrophilicity of the ions [15]. Similarly, the introduction of hydroxyl groups into the alkyl residue of ammonium ions in the 2D oxalate network [M^II^Cr^III^] (M^II^ = Mn^II^, Fe^II^, Co^II^) led to the formation of hydrophilic layers that enabled conductive pathways for protons [16]. The role of NH_4_^+^ in the formation of efficient hydrogen-bond networks in the 2D oxalate-bridged polymer of zinc(II), which indeed provides high proton conductivity, was investigated by exchanging ammonium ions, which are typical proton conductors, with non-hydrogen-bonding potassium ions [17,18]. The effect of crystallization water molecules on proton conduction of this coordination polymer has also been studied [19]. Furthermore, constructed 3D dimethylammonium-containing oxalate-bridged coordination polymers have shown high proton conductivity due to the formation of extensive hydrogen-bonded networks [8,20,21,22].

While high proton conductivity is often found in oxalate-based 2D and 3D assemblies, few examples emerge once the dimensionality lowers to one-dimensional (1D) polymers [7,8] such as [Fe(H_2_O)_2_(C_2_O_4_)]*_n_*, with coordinated water molecules forming a well-ordered 1D water nanoarray, allowing rational design of coordination polymers useful for solid electrolytes [23].

Here, we present an oxalate-bridged 1D coordination polymer {[NH(CH_3_)(C_2_H_5_)_2_][FeCl_2_(C_2_O_4_)]}*_n_* (**1**) that exhibits antiferromagnetic spin chains of the host iron(III) anions, [FeCl_2_(C_2_O_4_)]*_n_^n^*^−^, while the alkyl ammonium counterions and guest molecules give rise to high proton conductivity at room temperature (RT), which is quite unusual for 1D structural and magnetic arrangements. Strikingly, the proton conductivity of **1** increases by more than five orders of magnitude with increasing relative humidity (RH), achieving 2.70 × 10^−4^ (Ω·cm)^−1^ at 298 K and 93% RH. In addition, the title compound also shows great structural versatility with several single-crystal-to-single-crystal transformations induced by temperature.

## 2. Experimental

### 2.1. Materials and Physical Measurements 

All used chemicals were procured from commercial sources and used without further purification. The infrared spectrum was recorded using a KBr pellet by a Bruker Alpha-T spectrometer in the 4000–350 cm^−1^ range. Thermal properties were investigated from RT to 1073 K in synthetic air, with a Shimadzu DTG-60H analyzer (heating rate of 10 °C·min^−1^).

### 2.2. Synthetic Procedures

To an aqueous solution (10 mL) containing FeCl_3_ (0.1622 mg; 1 mmol) and H_2_C_2_O_4_·2H_2_O (0.1261 mg; 1 mmol), an amine, NCH_3_(C_2_H_5_)_2_ (2.2 mmol; 0.27 mL), was added. The reaction mixture was stirred for 10 min, after which it was filtered to remove a small amount of reddish precipitate, if formed. From a clear solution, yellow rod-like single crystals of {[NH(CH_3_)(C_2_H_5_)2][FeCl_2_(C_2_O_4_)]}*_n_* (**1**) formed in 2 weeks via slow evaporation. They were quickly washed using a small amount of ethanol and dried in air. The yield was 40%. Main IR absorptions bands (KBr, cm^−1^) were as follows: 3120–2815 (ν(NH) and ν(CH)), 1696 and 1608 (ν*_as_*(CO)), 1500–1375 (ν(CN)), 1348 and 1205 (ν*_s_*(CO)), 801 (δ(OCO)) [24].

### 2.3. Single-Crystal X-ray Structural Study

Single-crystal measurement of compound **HT-1** (243 K) was performed on an Oxford Diffraction Xcalibur Nova R diffractometer (Oxford Diffraction, Wroclaw, Poland) (*λ* = 1.54179 Å, microfocus Cu tube) equipped with an Oxford Instruments CryoJet liquid-nitrogen cooling device (Oxford Instruments, Abingdon, UK). Program package CrysAlis PRO was used for data reduction and numerical absorption correction [25]. Data collections for phases **LT-1** (100 K) and **MT-1** (180 K) were carried out on a Bruker D8 Venture (*λ* = 0.71073 Å, microfocus Mo tube) diffractometer (Bruker AXS, Madison, WI, USA) equipped with an Oxford Cryosystems Cryostream Series 700 liquid-nitrogen cooling device (Oxford Cryosystems, Oxford, UK). Data reduction and absorption correction were preformed using Bruker SAINT software package [26]. The structures were solved using SHELXS97 [27] and refined with SHELXL-2017 [28]. Models were refined using the full-matrix least squares refinement; all non-hydrogen atoms were refined anisotropically. Hydrogen atoms were located in a difference Fourier map and refined either as riding entities or a mixture of free restrained and riding entities. Undisordered structure **MT-1** was refined without restraints, while disordered cations in **LT-1** and **HT-1** structures were refined using rigid-body restraints (commands SIMU and DELU in SHELXS97); the C−C and C−N bonds were restrained to 1.54(2) and 1.45(2) Å, respectively. In addition, bond angles in cations in **LT-1** were restrained to C−C and C−N distances of 2.30(4) Å. Molecular geometry calculations were performed by PLATON [29,30], and molecular graphics were prepared using ORTEP-3 [31], and Mercury [32]. The crystallographic and refinement data for the structures reported in this work are given in Table 1.

### 2.4. Powder X-ray Structural Study

Temperature-induced structural changes were followed by in situ X-ray powder diffraction on a Bruker D8 Discover diffractometer (Bruker, Karlsruhe, Germany) equipped with LYNXEYE XE-T detector in Bragg–Brentano geometry (Bruker, Karlsruhe, Germany). Data were collected in the 2θ range 19.5–33° in the temperature range from 273 K to 80 K.

Crystal structures were refined using the Rietveld method in HighScoreXpert Plus (Version 4.5, March 2016). The thermal expansion coefficients were calculated from the refined unit-cell parameters obtained from the variable-temperature diffraction data. The axial thermal expansion coefficients along the principal axes were calculated using software PASCal [33].

### 2.5. Magnetization Study

Magnetic properties were studied on polycrystalline powder samples of **1** using a commercial MPMS 5 superconducting quantum interferometer device (SQUID) magnetometer (Quantum Design, San Diego, CA, USA). The pressed pellet was directly inserted into a measuring straw without any glue, such that the measured signal came only from the sample. The temperature dependence of the magnetization *M*(*T*) was measured in the temperature range 2–300 K in two modes; first, the sample was cooled in zero field and measured in the applied field *H* during heating (so-called ZFC curve), and then the sample was cooled in the same field in which the *M*(*T*) was measured afterward during heating (so-called FC curve). The magnetic hysteresis *M*(*H*) was measured at several stable temperatures in magnetic fields up to 5 T. No irreversibility was observed in both *M*(*T*) and *M*(*H*) measurements, indicating that there is no long-range ordering or spin freezing. For the calculation of the molar magnetic susceptibility *χ*, the measurement of *M*(*T*) in a field of 1000 Oe was used to ensure both a linear *M*(*H*) response and a sufficiently high signal to reduce the influence of noise, such that the calculated *χ*(*T*) = *M*(*T*)/*H* was a reliable physical function. A relatively broad maximum around 45 K in the *χ*(*T*) dependence points to the existence of an antiferromagnetic interaction and indicates the low-dimensional magnetic structure. In accordance with the chain structure of the iron(III) ions, the Fisher formula [34] for the susceptibility of magnetic chains consisting of large spins,

(1) $$\chi_{F}\left(T\right)=\frac{N_{A}g^{2}\mu_{B}{}^{2}S\left(S+1\right)}{3k_{B}T}\;\frac{1+u}{1-u},$$

where

(2) $$u=coth\left[\frac{JS\left(S+1\right)}{k_{B}T}\right]-\left[\frac{k_{B}T}{JS\left(S+1\right)}\right],$$

was used to fit the measured dependence. Here, *S* = 5/2 is large enough to ensure the validity of the Fisher formula. *J* is the exchange interaction between the neighboring spins used in the spin interaction Hamiltonian $H=-J\sum_{i}{\vec{S}}_{i}\cdot {\vec{S}}_{i+1}$, and other symbols have their usual meaning. Temperatures above 25 K were used in the fitting, because the Fisher function itself is limited to the temperatures not far below the maximum of the susceptibility (≈*J*). For the best-fitting curve, the obtained value for the intra-chain super-exchange interaction between the neighboring Fe^3+^ ions was *J* = (−7.58 ± 0.09) cm^−1^ (−10.9 ± 0.2) K, with *g* = (2.01 ± 0.01). The small rise in *χ* with cooling to the lowest temperatures came from the impurities. Fitting with the paramagnetic term added to the *χ*_F_ was not successful because of two possible effects: the Fisher function was not valid for the lowest temperatures, and the impurities might have been of some other origin but not paramagnetic. Comparing the shape of the *χ*(*T*) dependence with another *S* = 5/2 spin chain system [35], an impurity molar ratio well below 1% can be estimated. Although the fitted *χ*_F_ described the measured *χ*(*T*) dependence very well, extension of the Fisher function with the mean field correction was also tried in order to probe if there was some observable inter-chain interaction. The conclusion was that there were no detectable interactions between the chains, as could already be concluded from the good overlap of the Fisher function with the measured data.

### 2.6. Proton Conductivity Study

The conductivity of compound **1** was measured by impedance spectroscopy (Novocontrol Alpha-AN Dielectric Spectrometer, Novocontrol Technologies GmbH & Co. KG, Hundsangen, Germany) from 0.01 Hz to 1 MHz at 25 °C at different relative humidity values: 61% (air, ambient conditions), 75%, 84%, and 93%. The relative humidity inside the sample cell was obtained using saturated aqueous solutions of different salts: NaCl (RH = 75%), KCl (RH = 84%), and KNO_3_ (RH = 93%). In addition, the impedance spectra of compound **1** were measured in dry nitrogen at temperatures from −90 °C to 70 °C (temperature step: 10 °C) and in the frequency range from 0.01 Hz to 1 MHz. The measurements were performed on polycrystalline sample pressed into pellet of approximate thickness 0.8 mm. For the electrical contacts, gold electrodes (3.8 mm in diameter) were sputtered on the opposite surfaces of the pellet. The impedance spectra were analyzed by equivalent circuit modeling using the complex nonlinear least-squares fitting procedure (ZView software). From the values of electrical resistance (*R*) and electrode dimensions (A is the electrode area, and d is the sample thickness) the DC conductivity was calculated according to the following equation: *σ*_DC_ = *d*/(*A*·*R*).

## 3. Results and Discussion

### 3.1. Synthesis and Crystal Structures of Compound **1**

Yellow rod-shaped crystals of compound **1** were obtained via slow evaporation of an aqueous solution containing a mixture of FeCl_3_ (1 mmol), H_2_C_2_O_4_·H_2_O (1 mmol), and (C_2_H_5_)_2_(CH_3_)N (2.2 mmol). It is important to note that compound **1** could not be formed without excess amine. It remained stable up to 420 K, when the main mass loss occurred in two steps, followed by strong exothermic effects ending around 973 K (Figure S1).

**Figure 1:**
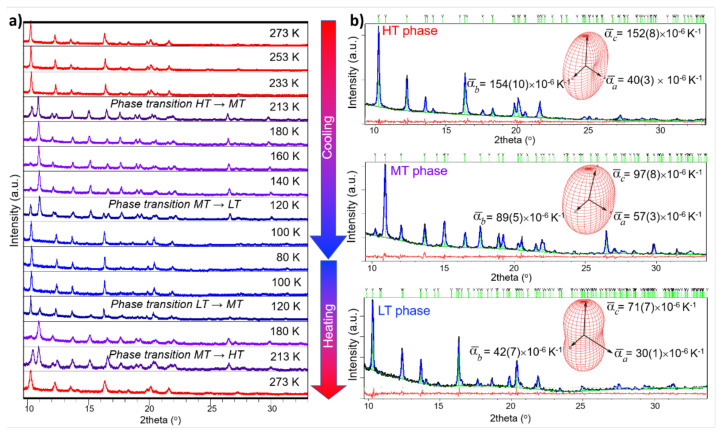
(**a**) In situ variable-temperature XRPD data of **1** collected in temperature range from 273 to 80 K during the cooling and heating run. Diffraction patterns of **HT-1**, **MT-1**, and **LT-1** are given in red, purple, and blue, respectively. (**b**) Rietveld refinements for **HT-1, MT-1**, and **LT-1** at *T* = 273, 180, and 100 K, respectively. Experimental data are given by a black line, the calculated pattern is shown in blue, and the red line represents the difference curve. Green vertical marks show the positions of Bragg reflections. The thermal expansivity indicatrix and corresponding thermal expansion coefficients for each phase are shown in insets.

During preliminary collection of single-crystal X-ray diffraction data of **1** at RT and later on at 100 K, a phase transformation was observed; to gain additional insight into temperature-induced structural transformation of **1**, in situ X-ray powder diffraction (XRPD) measurements were performed (Figure 1a). In situ variable-temperature XRPD revealed that compound **1** exhibits two reversible phase transitions: from the high-temperature phase **HT** to the mid-temperature phase **MT** at *T* ~213 K, and from the mid-temperature phase **MT** to the low-temperature **LT** phase at T ~120 K. The structures of **HT-1**, **MT-1**, and **LT-1** were determined by single-crystal X-ray diffraction at 243 K, 180 K, and 100 K, respectively. The crystal data and details of the data collections and refinements for the reported phases are summarized in Table 1.

Rietveld structure refinement of the powder data at each *T* was performed using initial structural models for **HT-1**, **MT-1**, and **LT-1** as determined by single-crystal diffraction (Figure 1b). The presence of the high-temperature polymorph **HT-1** was observed in the temperature range from 273 K to 233 K. At 213 K, sample contained both **HT-1** and **MT-1** phases (33.2 wt% **HT-1** and 66.8 wt% **MT-1**). The mid-temperature polymorph remained stable down to 120 K, when the transformation to the low-temperature phase **LT-1** occurred (78.5 wt% **MT-1** and 21.5 wt.% **LT-1**). The low-temperature polymorph remained stable down to 80 K. During the heating run, phase transitions were again observed at *T* ~120 K and *T* ~213 K; however, the quantitative composition was slightly different from that found during the cooling run, indicating a small hysteresis in the temperature of the phase transition (Figure 1).

**Figure 2:**
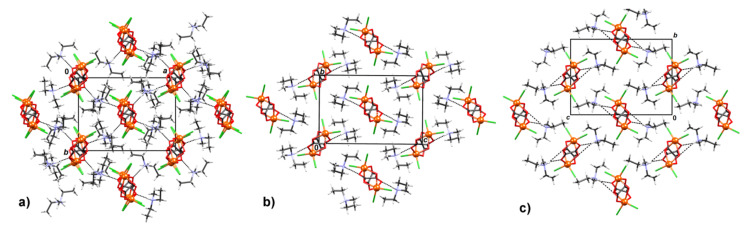
Packing of the [FeCl_2_(C_2_O_4_)]*_n_^n−^* chains in (**a**) **LT**, (**b**) **MT**, and (**c**) **HT** structures of coordination polymer {[NH(CH_3_)(C_2_H_5_)_2_][FeCl_2_(C_2_O_4_)]}*_n_* (**1**) viewed in the direction of chains.

Phases **HT** and **MT** crystallized in a *P*2_1_/*c* space group, while **LT** crystallized in *P*2_1_/*n* (Table 1), with all three comprising [FeCl_2_(C_2_O_4_)]*_n_^n^*^−^ chains packed in the same fashion and diethylmethylammonium cations (CH_3_)(C_2_H_5_)_2_NH^+^ located between them (Figure 2).

The temperature dependence of the unit-cell parameters is given in Table S1, while the thermal expansion behavior for each phase is represented by the thermal expansivity indicatrices shown as insets in Figure 1. It is noteworthy that all three polymorphs, but especially **HT-1**, exhibited large linear thermal expansion coefficients along the *b-* and *c*-directions (Figure 1b). Molecular crystals usually exhibit a thermal expansion coefficient in the range of 20 × 10^−6^ K^−1^, but large thermal expansions have recently been reported for (phenylazophenyl)palladium hexaflouracetyacetonate (260 × 10^−6^ K^−1^) [36], *N*′-2-propylidene-4-hydroxybenzohydrazide (360 × 10^−6^ K^−1^) [37], and a copper(II) complex with imidazoliums (346 × 10^−6^ K^−1^) [38].

**Figure 3:**
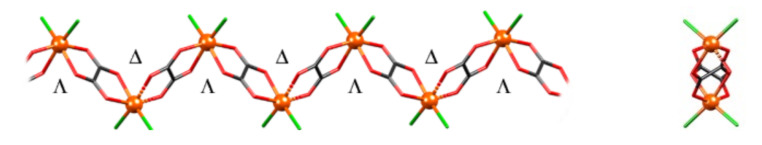
The [FeCl_2_(C_2_O_4_)]*_n_^n−^* chains in polymer **1** with indicated configuration of stereogenic iron(III) centers. A side-view of the chains is shown on the left, and a view along a chain is shown on the right.

Compound {[NH(CH_3_)(C_2_H_5_)_2_][FeCl_2_(C_2_O_4_)]}*_n_* (**1**) comprises infinite anionic chains [FeCl_2_(C_2_O_4_)]*_n_^n^*^−^ and diethylmethylammonium cations (C_2_H_5_)_2_(CH_3_)NH^+^. Each iron(III) center is coordinated by two oxalate ligands in a *cis* arrangement and two terminal Cl ligands occupying the remaining two positions (also in a *cis* fashion). Due to an approximate *C*_2_ symmetry of the coordination sphere, the Fe atoms act as stereogenic centers with alternating Λ- and Δ-configurations. Each oxalate ligand acts as a (bis)bidentate ligand bridging two Fe^3+^ ions with opposite configurations to form *zig-zag* chains (Figure 3); the Fe−O and Fe−Cl distances are similar to those previously reported [24,39,40,41,42]. Detailed geometric parameters describing the coordination of Fe ions in the three phases of compound **1** are given in Table S2.

**Figure 4:**
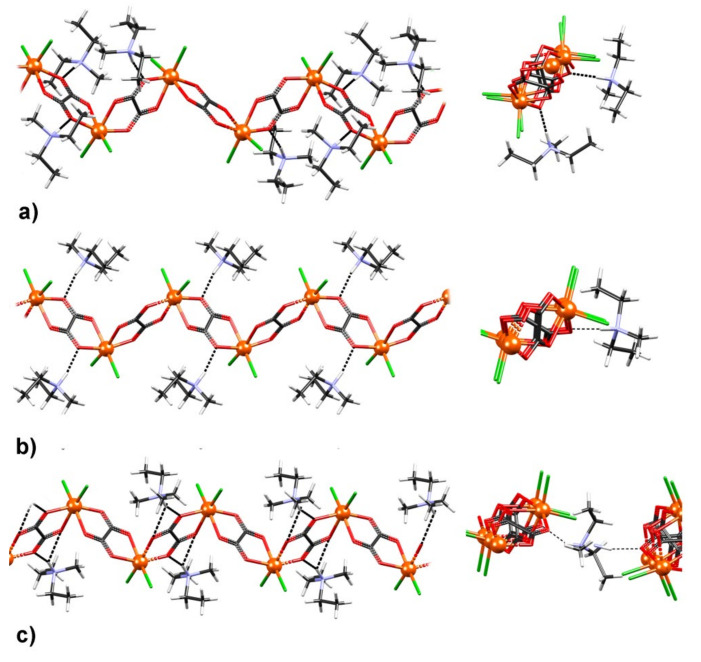
The [FeCl_2_(C_2_O_4_)]*_n_^n^*^−^ chains with hydrogen-bonded cations in (**a**) **LT-1**, (**b**) **MT-1**, and (**c**) **HT-1** structures. The inset shows a close-up view of hydrogen bonds formed by the cation.

The positions, as well as the arrangement of the cations acting as hydrogen bond donors toward the carbonyl oxygen atoms, were different in the three phases (Table S3). In the case of the **HT** structure, the cation was disordered over two positions; each donated a bifurcated hydrogen bond to a different [FeCl_2_(C_2_O_4_)]*_n_^n^^−^* chain (Figure 4c), whereas, in the **MT** structure, the cation was ordered and donated a single hydrogen bond (Figure 4b). The **LT** structure had two symmetry-independent cations (Z′ = 2) that had different conformations; in both, one ethyl group was disordered. Both cations served as donors of a single unbifurcated hydrogen bond (Figure 4a).

### 3.2. Magnetic and Charge Transport Properties of Compound **1**

**Figure 5:**
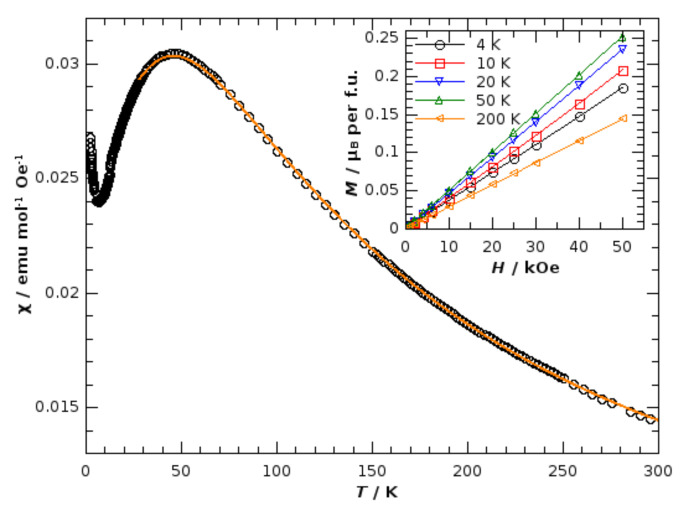
Temperature dependence of magnetic susceptibility, *χ*(*T*), measured in field of 1000 Oe. The orange solid line is the fitted curve. The inset shows the field dependence of magnetization, *M*(*H*), expressed in Bohr magnetons per formula unit, at different temperatures, with visual guidelines.

The chain structural ordering of iron(III) ions bridged by oxalate ligands was also confirmed by the temperature dependence of magnetic susceptibility, as shown in Figure 5. In agreement with the determined structure, the *χ*(*T*) is modeled using the Fisher formula [34]. Other relevant details related to the study of the magnetic properties of **1** are given in Section 2. The best-fitting curve was obtained for the antiferromagnetic exchange interaction *J* = −7.58(9) cm^−1^ between the neighboring iron(III) ions of spin 5/2 with *g* = 2.01(1) (Table S4). Linear *M*(*H*) curves were consistent with antiferromagnetic coupling along the chains, and their slopes at different temperatures reflect the *χ*(*T*) behavior. In contrast to some oxalate-bridged chains of iron(III) containing different counterions [24,39,40], which even exhibit a spontaneous magnetic order, no interaction between the chains was observed in **1**. In the case of **1,** the intra-chain interaction was stronger compared to most literature reports and approached the ideal Heisenberg spin-chain, as similarly found in [41].

**Figure 6:**
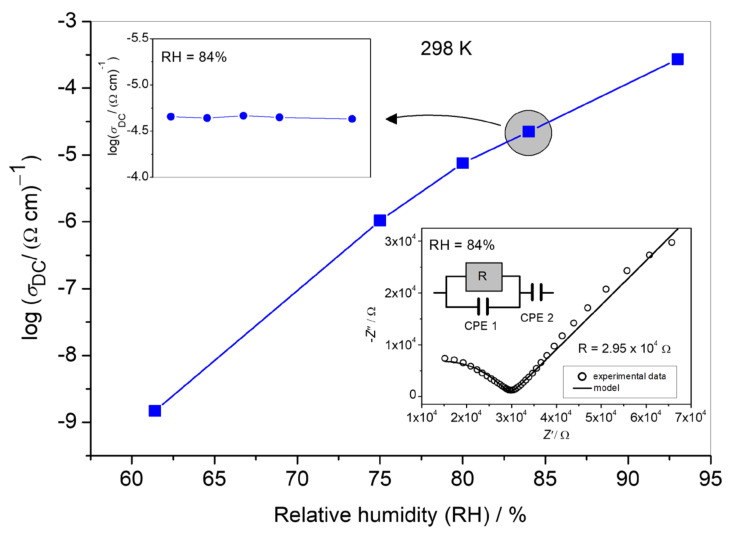
The proton conductivity of **1** as a function of relative humidity (RH) at room temperature. The inset in the left corner shows the time-dependent proton conductivity of **1** at 84% RH. The inset in the right corner shows the Nyquist plot of **1** measured at RH = 84%. The impedance semicircle corresponds to the bulk response of the sample, and low-frequency spur is related to the blocking of protons at the surface of the electrodes. The corresponding equivalent electrical circuit consists of a parallel combination of resistor and constant phase element (CPE 1), which models the bulk response, and an additional constant phase element (CPE 2) in series, which is related to the spur. The Nyquist plots of **1** in other RH conditions are given in Figure S3.

The proton conductivity of **1** was evaluated by impedance spectroscopy using a compacted pellet of the powder sample at various humidity rates. With the increase in RH from 61% to 93% at 298 K, the conductivity increases by more than five orders of magnitude and reaches the value of 2.70 × 10^−4^ (Ω·cm)^−1^, indicating the excellent humidity-sensing properties of **1** (Figure 6). The conductivity of **1** is several orders of magnitude higher than other 1D coordination polymers ({[Zn(C_10_H_2_O_8_)_0.5_(C_10_S_2_N_2_H_8_)]·2H_2_O]}*_n_* (1.33 × 10^−7^ (Ω·cm)^−1^ at RT and 95% RH) [43] and [Mn(dhbq)(H_2_O)_2_]*_n_* (4 × 10^−^^5^ (Ω·cm)^−^^1^ at RT and 98% RH; dhbq = 2,5-dihydroxy-1,4-benzoquinone]) [44], and it is rivaled only by 1D polymer [Fe(H_2_O)_2_(C_2_O_4_)]*_n_*, which achieved 1.3 × 10^−3^ (Ω·cm)^−1^ at 98% RH [23].

The strong dependence of conductivity upon humidity indicates that efficient proton transfer plays a crucial role in achieving the high conductivity values of **1** [7,8,9,10,11,12,13,14]. No change in the structure of **1** was observed after treatment with humidity, indicating the stability of compound **1** (Figure S2). Moreover, the time-dependent measurements show that **1** could maintain stable proton conductivity for a period of least 12 h, which is very beneficial for practical applications of this material (inset in Figure 6).

It is well established that water molecules have a pronounced effect on proton conductivity; as the number of waters increases, the conductivity increases due to the complex interpenetrating hydrogen bonding network, which enablse more efficient proton transfer [19]. Although not as pronounced as in the 3D frameworks, the best-performing 1D polymer [Fe(H_2_O)_2_(C_2_O_4_)]*_n_* also featured an interesting intermolecular assembly: two water molecules coordinated axially to a ferrous ion forming a 1D ordered array, in addition to the 1D chain composed of ferrous ions and oxalate. In this regard, it is particularly important to note that the coordination polymer **1** contains neither coordinated nor crystalline water.

The hydrophilic channels were filled with diethylmethylammonium cations, which were not mutually interconnected and, thus, did not form a significant hydrogen bonding network (Figure 4.) Nevertheless, the room-temperature proton conductivity of **1** is still quite comparable to that of [Fe(H_2_O)_2_(C_2_O_4_)]*_n_*, suggesting that the alkyl ammonium cations, which were only connected to the 1D coordination polymer via hydrogen bonds, can also facilitate fast and efficient proton transfer, leading to the observed high proton conductivity in **1**.

Moreover, it should be noted that the proton conductivity of compound **1** is comparable to that of metal–organic frameworks (MOFs), which typically lies in the range ∼10^−^^2^−10^−^^4^ (Ω·cm)^−1^ [7,14]. Furthermore, it is close in value to the proton conductivity of covalent organic frameworks (COFs) with the best proton conductive performance of ∼10^−^^1^−10^−3^ (Ω·cm)^−1^ [45].

Interestingly, the phase transition between the **HT-1** and **MT-1** phases was also evident in the temperature dependence of the conductivity measured in dry nitrogen, where two distinct ranges were observed: below 213 K characterized by a lower activation energy for the conductivity, *E*_a_ = 0.42 eV, and above this temperature, where the activation energy was higher, *E*_a_ = 0.62 eV (Figure S4). It can be concluded that the as-prepared sample (**HT-1**) has a low intrinsic proton conductivity under ambient conditions, which is thermally activated with an activation energy of 0.62 eV, indicating a vehicle mechanism related to the diffusion of protons combined with the solvent acting as a vehicle. This is to be expected considering that the hydrogen-bonding array within the channel is not very complex and, thus, proton hopping is reduced. By lowering the temperature, the presence of the phase **MT** (Figure 1) decreases the activation energy, approaching the Grotthus mechanism [7,9]. It is likely that the different binding of the cations to the oxalate chains through hydrogen bonds affects the observed changes in the activation energy (Figure 4 and Table S3) of proton conductivity, indicating that the structural arrangement of **MT-1** is more suitable for proton transport.

## 4. Conclusions

In summary, we reported an oxalate-bridged 1D material {[NH(CH_3_)(C_2_H_5_)_2_][FeCl_2_(C_2_O_4_)]}*_n_* that exhibits great structural flexibility demonstrated by the appearance of three different polymorphic phases that possess additional multifunctional features; the compound consists of antiferromagnetic spin chains with strong exchange interactions but also shows excellent humidity-sensing properties and high proton conductivity. These studies show that the incorporation of a relatively simple yet quite flexible alkyl-substituted ammonium derivative as a counterion can lead to an emergence of important intrinsic properties of materials that are attractive for various applications.

## Figures and Tables

**Table 1:** Crystallographic data and structure refinement details for variable-temperature X-ray diffraction of compound {[NH(CH_3_)(C_2_H_5_)_2_][FeCl_2_(C_2_O_4_)]}*_n_* (**1**).

Compound	LT-1 (100 K)	MT-1 (180 K)	HT-1 (243 K)
Empirical formula	C_7_H_14_Cl_2_FeNO_4_	C_7_H_14_Cl_2_FeNO_4_	C_7_H_14_Cl_2_FeNO_4_
Formula wt. (g·mol^−1^)	302.94	302.94	302.94
Color	yellow	yellow	yellow
Crystal dimensions/mm	0.18 × 0.09 × 0.07	0.20 × 0.18 × 0.09	0.20 × 0.18 × 0.08
Space group	*P*2_1_/*n*	*P*2_1_/*c*	*P*2_1_/*c*
*a* (Å)	14.962(3)	8.720(2)	8.5125(8)
*b* (Å)	10.791(2)	9.762(2)	10.7755(14)
*c* (Å)	16.944(3)	14.830(3)	14.9703(16)
*α* (°)	90	90	90
*β* (°)	105.96(3)	95.43(3)	104.798(9)
*γ* (°)	90	90	90
Z	8	4	4
*V* (Å^3^)	2630.1(9)	1256.8(5)	1327.6(3)
*D*_calc_ (g·cm^−3^)	1.530	1.601	1.516
*λ* (Å)	0.71073 (Mo*K*α)	0.71073 (Mo*K*α)	1.54179 (Cu*K*α)
*μ* (mm^−1^)	1.547	1.618	12.807
*Θ* range (°)	2.26–27.57	2.35–26.53	5.12–74.32
*T* (K)	100(2)	180(2)	243(2)
Diffractometer type	D8 Venture	D8 Venture	Xcalibur Nova
Range of *h*, *k*, *l*	19 < *h* < 18; 13 < *k* < 13; −21 < *l* < 22	−10 < *h* < 10; −12 < *k* < 10; −18 < *l* < 17	−10 < *h* < 6; −13 < *k* < 13; −18 < *l* < 17
Reflections collected	58295	10852	5948
Independent reflections	6028	2595	2423
Observed reflections (*I*≥2*σ*)	4917	1937	1440
Absorption correction	None	None	Multi-scan
*T*_min_, *T*_max_	−	−	0.21266; 1.0000
R_int_	0.0597	0.0590	0.0542
R (F)	0.0349	0.0416	0.0873
R_w_ (F^2^)	0.0904	0.1155	0.2990
Goodness of fit	1.043	1.066	1.010
H atom treatment	Mixed	Constrained	Constrained
No. of parameters	315	139	147
No. of restraints	91	0	77
Δρ_max_, Δρ_min_ (eÅ^–3^)	0.890; −0.520	0.570; −0.467	0.444; −0.381

## Data Availability

The data presented in this study are available in the Supplementary Materials.

## Supplementary Materials

The following are available online at https://www.mdpi.com/article/10.3390/ma14195543/s1: Figure S1. The TG and DTA curves (heating and cooling) of compound **1** measured in synthetic air; Table S1. Temperature dependence of the unit-cell parameters for **HT-1**, **MT-1**, and **LT-1** phases of coordination polymer {[NH(CH_3_)(C_2_H_5_)_2_][FeCl_2_(C_2_O_4_)]}*_n_* (**1**); Table S2. Selected distances (Å) and angles (°) for the coordination sphere of the Fe ions in three phases of compound {[NH(CH_3_)(C_2_H_5_)_2_][FeCl_2_(C_2_O_4_)]}*_n_* (**1**). Symmetry operators: (*i*) 1 − *x*, 1 − *y*, 2 − *z*; (*ii*) 1 − *x*, 1 − *y*, 1 − *z*; (*iii*) 2 − *x*, 1 − *y*, 1 – *z;* Table S3. Geometric parameters of hydrogen bonds (Å, °) for three phases of compound {[NH(CH_3_)(C_2_H_5_)_2_][FeCl_2_(C_2_O_4_)]}*_n_* (**1**); Table S4. Distances between oxalate-bridged iron(III) ions in compound **1**. Symmetry operators on the second Fe ion: (*i*) 1 − *x*, 1 − *y*, 2 − *z*; (*ii*) − *x*, − *y*, − *z*; (*iii*) 2 − *x*, 1 − *y*, 1 − *z*; (*iv*) 1 − *x*, 1 − *y*, 1 – *z*; Figure S2. The X-ray powder diffraction patterns of coordination polymer **1** (a) prior to and (b) after the humidity treatment; **Figure S3**. Nyquist plot of **1** at 298 K and (a) 61% (ambient), (b) 75%, (c) 80%, and (d) 93% relative humidity (RH). The circles denote experimental impedance data and lines correspond to model impedance obtained by equivalent circuit modeling. The equivalent circuit for all impedance spectra consists of a parallel combination of resistor and constant phase element (CPE 1), which represents the bulk response of the sample, and an additional constant phase element (CPE 2), which models the low-frequency spur arising from the electrode polarization; Figure S4. Conductivity as a function of reciprocal temperature for compound **1**. Solid lines represent least-squares linear fit to experimental data for the two temperature ranges: from −90 °C to −60 °C (**MT-1**) and from −60 °C to 70 °C (**HT-1**). CCDC 2055768−2055770 contains the supplementary crystallographic data for this paper. These data can be obtained free of charge via http://www.ccdc.cam.ac.uk/conts/retrieving.html (accessed on 22 September 2021) (or from the CCDC, 12 Union Road, Cambridge CB2 1EZ, UK; Fax: +44-1223-336033; E-mail: deposit@ccdc.cam.ac.uk).
