# Supplementary material for: Humidity-Sensing Properties of an 1D Antiferromagnetic Oxalate-Bridged Coordination Polymer of Iron(III) and Its Temperature-Induced Structural Flexibility
Source: Materials (Basel). 2021 Sep 24;14(19):5543. doi: 10.3390/ma14195543 (PMC8509436; doi:10.3390/ma14195543)
Supplement: Supplementary file 1 [file materials-14-05543-s001.zip › materials-1386596-supplementary.pdf]

materials

Supplementary Materials

Humidity Sensing Properties of an 1D Antiferromagnetic Oxalate-Bridged Coordination Polymer of Iron(III) and its Temperature-Induced Structural Flexibility

Sanja Burazer [1], Krešimir Molčanov [1], Ana Šantić [1], Teodoro Klaser [2], Emmanuel Wenger [3], Damir Pajić [2], Zvonko Jagličić [4,5], Jasminka Popović [1] and Marijana Jurić [1,*]

[1] Ruđer Bošković Institute, Bijenička cesta 54, 10000 Zagreb, Croatia
[2] Department of Physics, Faculty of Science, University of Zagreb, Bijenička cesta 32, 10000 Zagreb, Croatia
[3] CRM2 CNRS, UMR 7036, Institut Jean Barriol, Université de Lorraine, BP 70239, Vandoeuvre-lès-Nancy, France
[4] Institute of Mathematics, Physics and Mechanics, Jadranska 19, 1000 Ljubljana, Slovenia
[5] Faculty of Civil and Geodetic Engineering University of Ljubljana Jamova 2, 1000 Ljubljana, Slovenia

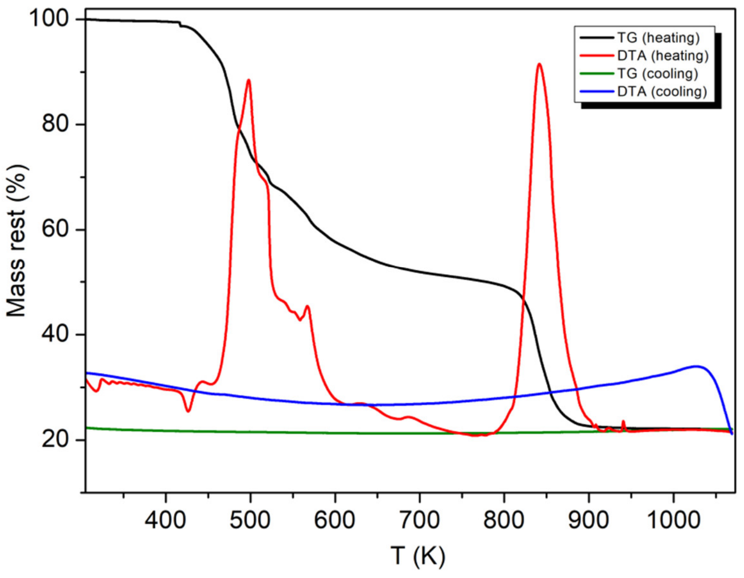

Figure S1. The TG and DTA curves (heating and cooling) of compound **1** measured in synthetic air.

Table S1. Temperature dependence of the unit-cell parameters for **HT-1**, **MT-1** and **LT-1** phases of coordination polymer $\{[NH(CH_3)(C_2H_5)_2][FeCl_2(C_2O_4)]\}_n$ (**1**).

T (K)	*a* (Å)	*b* (Å)	*c* (Å)	*β* (°)
		**HT-1**		
273	8.508(1)	10.752(2)	14.948(3)	104.82(1)
253	8.496(2)	10.722(3)	14.905(2)	104.78(2)
233	8.490(2)	10.697(2)	14.872(3)	104.77(3)
213	8.481(3)	10.652(2)	14.807(3)	104.67(2)
		**MT-1**		
213	8.714(1)	9.748(2)	14.802(3)	95.44(1)
180	8.701(2)	9.728(3)	14.770(3)	95.41(2)
160	8.688(2)	9.704(2)	14.738(3)	95.39(3)
140	8.675(2)	9.686(3)	14.699(3)	95.35(2)
		**LT-1**		
120	14.972(3)	10.801(2)	16.951(3)	105.94(2)
100	14.959(4)	10.799(2)	16.938(3)	105.91(1)
80	14.948(3)	10.783(3)	16.914(3)	105.88(2)

Table S2. Selected distances (Å) and angles (°) for the coordination sphere of the Fe ions in three phases of compound $\{[NH(CH_3)(C_2H_5)_2][FeCl_2(C_2O_4)]\}_n$ (**1**). Symmetry operators: *i*) 1 − *x*, 1 − *y*, 2 − *z*; *ii*) 1 − *x*, 1 − *y*, 1 − *z*; *iii*) 2 − *x*, 1 − *y*, 1 − *z*.

LT (Fe1)		LT (Fe2)		MT		LT	
Fe1-O5	2.0387(17)	Fe2-O3	2.0369(17)	Fe1-O1	2.020(2)	Fe1-O3	2.041(5)
Fe1-O2	2.0745(17)	Fe2-O7	2.0659(16)	Fe1-O3	2.0775(19)	Fe1-O1	2.048(5)
Fe1-O6^{i}	2.1454(18)	Fe2-O4	2.1761(16)	Fe1-O2^{iii}	2.151(2)	Fe1-O4	2.156(6)
Fe1-O1	2.1670(17)	Fe2-O8^{ii}	2.1880(18)	Fe1-O4^{ii}	2.153(2)	Fe1-O2	2.160(5)
Fe1-Cl2	2.2659(8)	Fe2-Cl4	2.2532(8)	Fe1-Cl2	2.2386(11)	Fe1-Cl1	2.229(4)
Fe1-Cl1	2.2409(9)	Fe2-Cl3	2.2412(9)	Fe1-Cl1	2.2442(11)	Fe1-Cl2	2.234(3)
O5-Fe1-O2	158.30(7)	O3-Fe2-O7	157.80(7)	O1-Fe1-O3	160.68(9)	O3-Fe1-O1	157.7(2)
O5-Fe1-O6^{i}	79.51(7)	O3-Fe2-O4	79.05(7)	O1-Fe1-O2^{iii}	78.86(8)	O3-Fe1-O4	78.1(2)
O5-Fe1-O1	84.21(7)	O3-Fe2-O8^{ii}	84.41(7)	O1-Fe1-O4^{ii}	88.35(9)	O3-Fe1-O2	84.12(19)
O5-Fe1-Cl2	94.09(6)	O3-Fe2-Cl4	99.84(6)	O1-Fe1-Cl2	96.47(7)	O3-Fe1-Cl1	99.90(17)
O5-Fe1-Cl1	101.80(6)	O3-Fe2-Cl3	95.90(6)	O1-Fe1-Cl1	99.87(8)	O3-Fe1-Cl2	94.64(18)
O2-Fe1-O6^{i}	85.08(7)	O7-Fe2-O4	83.90(7)	O3-Fe1-O2^{iii}	85.07(8)	O1-Fe1-O4	85.0(2)
O2-Fe1-O1	77.80(7)	O7-Fe2-O8^{ii}	78.06(7)	O3-Fe1-O4^{ii}	77.99(8)	O1-Fe1-O2	78.27(18)
O2-Fe1-Cl2	97.93(6)	O7-Fe2-Cl4	94.13(6)	O3-Fe1-Cl2	97.08(7)	O1-Fe1-Cl1	94.63(17)
O2-Fe1-Cl1	93.57(6)	O7-Fe2-Cl3	97.53(6)	O3-Fe1-Cl1	91.03(7)	O1-Fe1-Cl2	98.70(17)
O6^{i}-Fe1-O1	78.62(7)	O4-Fe2-O8^{ii}	77.52(7)	O2^{iii}-Fe1-O4^{ii}	78.80(8)	O4-Fe1-O2	78.3(2)
O6^{i}-Fe1-Cl2	167.44(6)	O4-Fe2-Cl4	166.64(6)	O2^{iii}-Fe1-Cl2	167.81(7)	O4-Fe1-Cl1	90.50(18)
O6^{i}-Fe1-Cl1	90.87(6)	O4-Fe2-Cl3	89.66(6)	O2^{iii}-Fe1-Cl1	91.11(7)	O4-Fe1-Cl2	166.38(18)
O1-Fe1-Cl2	90.06(6)	O8^{ii}-Fe2-Cl4	89.74(6)	O4^{ii}-Fe1-Cl2	89.87(7)	O2-Fe1-Cl1	167.2(2)
O1-Fe1-Cl1	166.84(6)	O8^{ii}-Fe2-Cl3	165.55(6)	O4^{ii}-Fe1-Cl1	165.59(7)	O2-Fe1-Cl2	89.54(17)
Cl2-Fe1-Cl1	101.06(4)	Cl4-Fe2-Cl3	103.45(4)	Cl2-Fe1-Cl1	100.83(5)	Cl1-Fe1-Cl2	102.19(14)

Table S3. Geometric parameters of hydrogen bonds (Å, °) for three phases of. compound $\{[NH(CH_3)(C_2H_5)_2][FeCl_2(C_2O_4)]\}_n$ (**1**).

	D–H / Å	H···*A* / Å	*D*···*A* / Å	*D*–H···*A* / º	Symm. op. on *A*
**LT-1**					
N1–H1···O7	1.00	1.90	2.896(3)	178	*x*, *y*, *z*
N2–H2···O2	1.00	1.86	2.852(3)	170	*x*, *y*, *z*
**MT-1**					
N1–H1···O3	1.00	1.90	2.888(4)	169	*x*, *y*, *z*
**HT-1**					
N1B–H1B···O1	0.98	1.97	2.95(3)	173	*x*, *y*, 1 + *z*
N1A–H1A···O3	0.98	1.92	2.86(3)	159	*x*, 1/2 − *y*, 1/2 + *z*

Table S4. Distances between oxalate-bridged iron(III) ions in compounds **1**. Symmetry operators on the second Fe Ion: *i*) 1 − *x*, 1 − *y*, 2 − *z*; *ii*) − *x*, − *y*, − *z*; *iii*) 2 − *x*, 1 − *y*, 1 − *z*; *iv*) 1 − *x*, 1 − *y*, 1 − *z*.

	**LT-1**	**MT-1**	**HT-1**
Fe1···Fe1	5.461(2)^{i}	5.523(2)^{iv}	5.481(2)^{iv}
Fe1···Fe1		5.467(2)^{iii}	5.492(2)^{iii}
Fe1···Fe2	5.507(2)		
Fe2···Fe2	5.555(2)^{ii}		

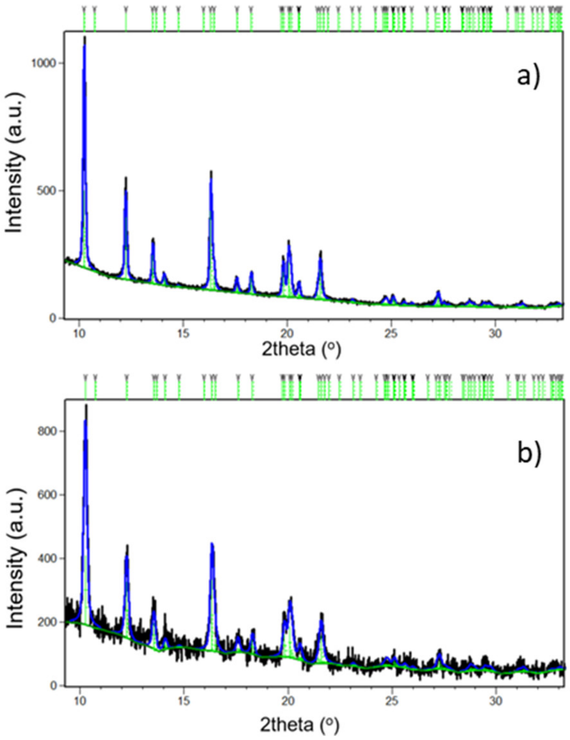

Figure S2. The X-ray powder diffraction patterns of coordination polymer **1**: a) prior to and b) after the humidity treatment.

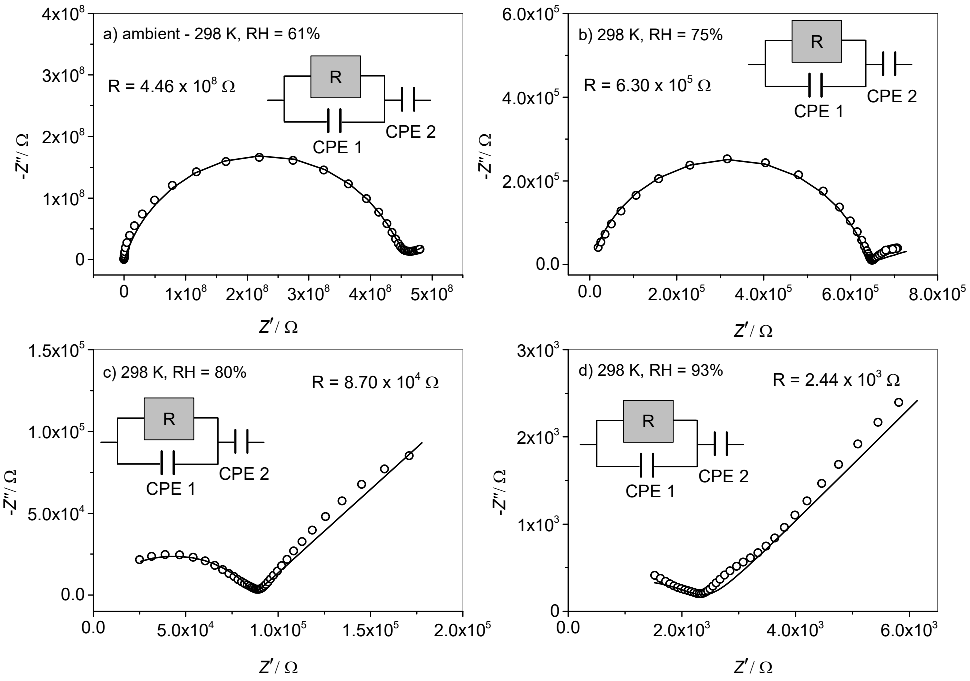

Figure S3. Nyquist plot of **1** at 298 K and (a) 61% (ambient), (b) 75%, (c) 80% and (d) 93% of relative humidity (RH). The circles denote experimental impedance data and lines correspond to model impedance obtained by equivalent circuit modelling. The equivalent circuit for all impedance spectra consists of parallel combination of resistor and constant phase element (CPE 1), which represents bulk response of the sample, and additional constant phase element (CPE 2) which models low-frequency spur arising from the electrode polarisation.

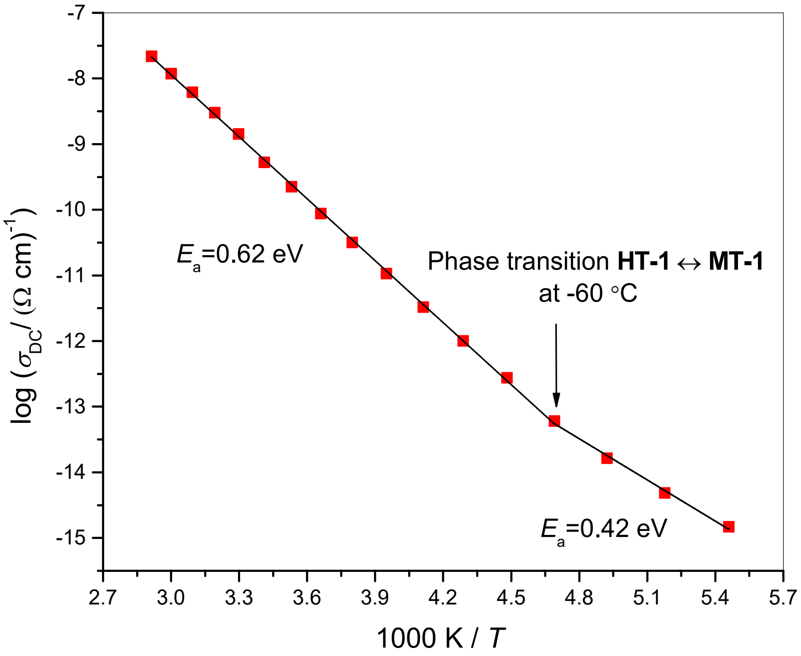

Figure S4. Conductivity as a function of reciprocal temperature for compound **1**. Solid lines represent least-squares linear fit to experimental data for the two temperature ranges: from −90 °C to −60 °C (**MT-1**) and from −60 °C to 70 °C (**HT-1**).
